# The life and times of endogenous opioid peptides: Updated understanding of synthesis, spatiotemporal dynamics, and the clinical impact in alcohol use disorder

**DOI:** 10.1016/j.neuropharm.2022.109376

**Published:** 2022-12-11

**Authors:** Elyssa B. Margolis, Madelyn G. Moulton, Philip S. Lambeth, Matthew J. O’Meara

**Affiliations:** a UCSF Weill Institute for Neurosciences, Department of Neurology, University of California, San Francisco, CA, USA; b Neuroscience Graduate Program, University of California, San Francisco, CA, USA; c Department of Computational Medicine and Bioinformatics, University of Michigan, Ann Arbor, MI, 48109, USA

## Abstract

The opioid G-protein coupled receptors (GPCRs) strongly modulate many of the central nervous system structures that contribute to neurological and psychiatric disorders including pain, major depressive disorder, and substance use disorders. To better treat these and related diseases, it is essential to understand the signaling of their endogenous ligands. In this review, we focus on what is known and unknown about the regulation of the over two dozen endogenous peptides with high affinity for one or more of the opioid receptors. We briefly describe which peptides are produced, with a particular focus on the recently proposed possible synthesis pathways for the endomorphins. Next, we describe examples of endogenous opioid peptide expression organization in several neural circuits and how they appear to be released from specific neural compartments that vary across brain regions. We discuss current knowledge regarding the strength of neural activity required to drive endogenous opioid peptide release, clues about how far peptides diffuse from release sites, and their extracellular lifetime after release. Finally, as a translational example, we discuss the mechanisms of action of naltrexone (NTX), which is used clinically to treat alcohol use disorder. NTX is a synthetic morphine analog that non-specifically antagonizes the action of most endogenous opioid peptides developed in the 1960s and FDA approved in the 1980s. We review recent studies clarifying the precise endogenous activity that NTX prevents. Together, the works described here highlight the challenges and opportunities the complex opioid system presents as a therapeutic target.

## 1. Introduction

Drugs targeting opioid GPCRs remain the most effective treatments for acute intense pain (Bagley and Ingram, 2020; Corder et al., 2018; Lindsay et al., 2021) and alcohol use disorder (AUD), and are in clinical trials to treat major depressive disorder (MDD; Krystal et al., 2020; Pizzagalli et al., 2020). Yet after decades of “rational” development, available opioid-targeted therapies continue to have substantial liabilities. Mu opioid receptor (MOR) agonist drugs cause life threatening depression of respiration (Aghabiklooei et al., 2013; Gerak et al., 2018) and disruption of gut motility (Holzer, 2009). Continuous use can lead to rapid development of tolerance and risks development of opioid use disorder (OUD; Marie, 2019). OUD-related costs in the US exceed $1B/year (Luo et al., 2021) and accidental toxic opioid poisonings now contribute to over 70,000 deaths/year. A major scientific challenge in overcoming these liabilities is that *in vitro* systems, cell lines that overexpress the receptor of interest and a reporter signaling pathway, largely fail to predict clinical outcomes. For instance, full MOR agonists should produce reward and we expect all such compounds will be self-administered in animal models. Yet, a cyclized variant of endomorphin 1 (EM-1), ZH853, which has similar *in vitro* activities to other full MOR agonists, does not support self administration in rodent studies (Zadina et al., 2016). *In vivo* models also often lack predictive power. To explain this and other unexpected observations, a range of mechanistic hypotheses and ligand features have been proposed, including biased agonism, partial agonism, ion-dependence, heteromeric complexes, alternative splicing, G protein selectivity, and polypharmacology. However, fundamentally, a complete explanation must recognize how opioid drugs interact with the endogenous opioid system. In turn, this requires a thorough characterization of the regulation and function of endogenous opioid peptides. Understanding how the endogenous opioid peptides act at the opioid receptors may also improve ligand targeting because the endogenous systems have evolved together and are highly conserved throughout vertebrate evolution (Abbadie et al., 2002; Larhammar et al., 2015; Li et al., 1996; Stevens, 2009).

Here we summarize basic facts, recent developments, and knowledge gaps about how the at least 26 endogenous opioid peptides are regulated and function in the central nervous system. We present information on the synthesis of the endogenous opioid peptides and what is known regarding how they interact with the different members of the opioid receptor family. We describe examples of different modes of subcellular neuronal distributions of the peptide precursors and putative signaling peptides, and how this relates to potential release sites and receptor target localization. We then describe what these distributions might mean for how we conceptualize the spatiotemporal dynamics of opioid neuromodulation in the brain and thus highlight some remaining fundamental questions about the properties and functions of the endogenous opioid peptide system. Finally, to illustrate the clinical significance of understanding and targeting the endogenous opioid system, we describe how naltrexone’s (NTX) interference with endogenous opioid peptide signaling treats AUD. We review NTX’s clinical efficacy and some of the subsequent research aimed at learning more about the underlying biological phenomena.

## 2. Basics on the peptides

In 1975, Hughes and colleagues first identified endogenously produced peptides that activate opioid receptors, the pentapeptides met-enkephalin (Tyr-Gly-Gly-Phe-Met; YGGFM; MENK) and leu-enkephalin (Tyr-Gly-Gly-Phe-Leu; YGGFL; LENK; Hughes et al., 1975). Subsequent studies identified at least 28 additional endogenous peptides that activate the opioid receptors; many of these are longer peptide fragments that include the Tyr-Gly-Gly-Phe motif. Early pharmacology studies proposed three opioid receptors: “mu” for its response to morphine (MOR), “kappa” for its response to ketazocine (KOR), and “delta” for the physiological response of the mouse vas deferens assay (DOR; Lord et al., 1977; Martin et al., 1976). Agonist effects at these three receptors were all blocked by NTX, thus they were considered the family of opioid receptors (Takemori and Portoghese, 1984). Later gene sequencing studies identified a fourth receptor, opioid-like receptor 1/nociceptin receptor with approximately 60% amino acid sequence homology with the other 3 receptors (Mollereau et al., 1994, reviewed in Toll et al., 2016). Gene studies led to the identification of most of the precursors for the active endogenous opioid peptides even before the receptors were cloned, except for nociceptin (Cox, 1982; Meunier et al., 1995; Reinscheid et al., 1995). Here we provide a very brief overview of the known sources of the identified endogenous opioid peptides from a precursor perspective. For a detailed description of the biochemistry of how proenkephalin (PENK), prodynorphin (PDYN), and proopiomelanocortin (POMC) are cleaved and which enzymes are involved in their processing please see (Fricker et al., 2020).

**Fig. 1.**
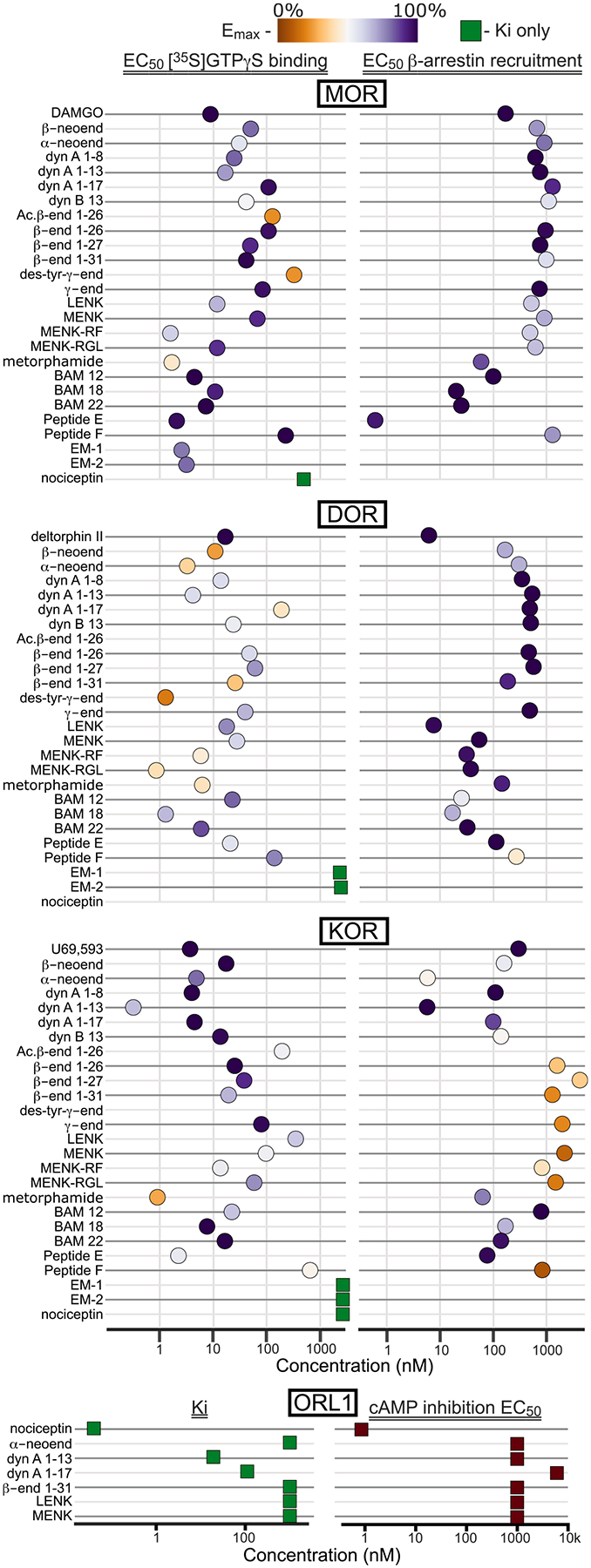
Binding and activity of endogenous opioid peptides at MOR, DOR, KOR, and ORL1. For MOR, DOR, and KOR, x-axis position is the EC_50_ concentration for G protein signaling (left) or β-arrestin signaling (right). Color indicates efficacy at 10 μM concentration compared to full agonists DAMGO (at MOR), deltorphin II (at DOR), or U69,593 (at KOR). Where signaling data are not available, Ki is graphed. For ORL1, only Ki and EC_50_ of cAMP inhibition were available. Data reported in (Butour et al., 1997; Gomes et al., 2020; Judd et al., 2003; Lapalu et al., 1998; Liu et al., 2017; Reinscheid et al., 1996; Sasaki et al., 2006; Wang et al., 2012).

For most of the identified endogenous ligands, the differences in their binding affinities to MOR, DOR, and KOR are not remarkably different (Fig. 1; Gomes et al., 2020; Kosterlitz, 1987). For signaling there is an added layer of complexity. Several recent studies demonstrate that although different peptides carry an identical opioid consensus sequence of Tyr-Gly-Gly-Phe (the ‘address’ terminus that determines binding), the additional amino acids on the non-address terminus (the ‘message’) determine the preferred signaling pathway of the peptide-receptor complex consistent with the signaling bias model (Gomes et al., 2020; Portoghese, 1989; Schwyzer, 1977; Thompson et al., 2015, 2016). Signaling bias may take the form of preference for a specific signaling pathway such as G protein versus β-arrestin dependent signaling (Fig. 1) or preference for trafficking of receptors for recycling or degradation. Thus, the identity of the released peptide is critical to understanding the downstream effects of opioid neurotransmission, beyond simply which receptor it is binding. For additional information on the translational potential of selective targeting of the different members of the opioid receptor family we recommend (Dalefield et al., 2022; Khan et al., 2022; Parker et al., 2020; Pradhan et al., 2011; Witkin et al., 2014). It is also important to note that non-opioid receptor activities have been identified for some opioid peptides as well (reviewed in Kaczyńska and Wojciechowski, 2021; Palmer et al., 2021). Next we describe the form and function for the five major classes of endogenous opioid peptides, focusing on knowledge gaps and recent developments.

### 2.1. Proenkephalin

**Fig. 2.**
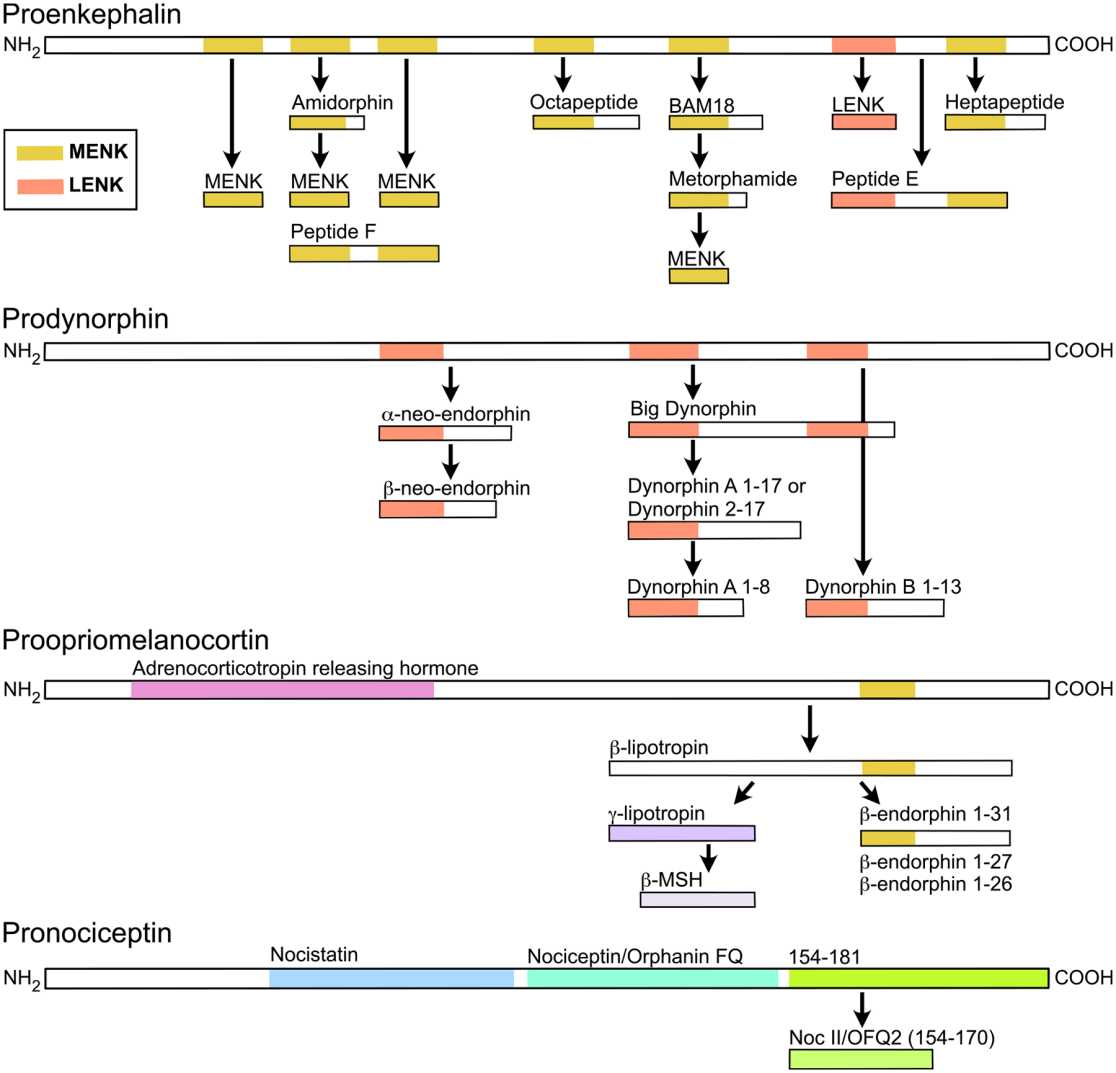
Peptide precursors and products of proenkephalin, prodynorphin, proopiomelanocortin, and pronociceptin. Organization of PENK is based on mouse and bovine studies (Fricker et al., 2020; Johanning et al., 1998; Jones et al., 1980). PDYN organization is based on human (reviewed in Cahill et al., 2021). POMC is based on mouse studies (reviewed in Fricker et al., 2020; Millington, 2007). PNOC is based on rat studies (Rossi et al., 2002). Illustration not to scale.

Although it is often thought that the signaling response generated from PENK peptides occurs through MOR activation, for some of these peptides their highest affinities are at other opioid receptors. The PENK peptide encodes 6 copies of MENK, 4 of which are predicted to be cleaved, and 1 copy of LENK (Fig. 2; Yoshikawa et al., 1984). Longer peptides that contain the enkephalin sequence can be cleaved from PENK and also signal at opioid receptors, including MENK-arg-phe (MENK-RF/heptapeptide), MENK-arg-gly-leu (MENK-RGL/octapeptide), BAM-18, metorphamide, amidorphin, peptide E, and peptide F (Fig. 2; Fricker et al., 2020). It remains unknown whether MENK and LENK are the primary signaling molecules generated from PENK or if the longer peptide fragments also participate in neurotransmission.

### 2.2. Prodynorphin

PDYN is the source of dynorphin peptides, which are broadly thought to underlie aversive signals through activation of the KOR. This precursor was first described as Proenkephalin B, due to its similarity to PENK (Chavkin et al., 1982; James et al., 1982). Peptides generated from PDYN include dynorphin 1–8, dynorphin-A 1–17, dynorphin-B 1–13, dynorphin 2–17, big dynorphin, and α- and β-neo-endorphin (Fig. 2). In addition to activating KORs, dynorphins do also appear to have relatively high affinity for MOR and DOR (Fig. 1; Gomes et al., 2020). For instance, the affinities of dynorphin A 1–8, 1–17, and dynorphin B 1–13 are within an order of magnitude of MENK at MOR (Gomes et al., 2020). For β-neo-endorphin, binding affinity and potency at KOR and DOR are not distinguishable (Gomes et al., 2020; Mansour et al., 1995). On the other hand, in recent studies the *in vivo* impacts of selective stimulation of *Pdyn* neurons have been blocked by selective KOR antagonists (e.g., Abraham et al., 2021; Al-Hasani et al., 2015). Such selectivity may also be facilitated by neural structural and organizational features described below.

Interestingly, the address end of these PDYN peptides is the LENK sequence; *Pdyn* contains 3 copies of the LENK sequence plus the appropriate cleavage sites to generate the pentapeptide (Fig. 2). It remains to be determined if PENK, PDYN, or an unidentified precursor gives rise to LENK destined for signaling. Evidence supporting the possibility that LENK is a product of PENK alone includes early *ex vivo* release studies measuring MENK and LENK release from acute striatal brain slice preparations, which contain both *Penk* and *Pdyn* neurons. Here the peptides were detected at ratios of between 3:1 and 4:1, consistent with the predicted ratio of MENK to LENK synthesized from PENK alone (Henderson et al., 1978). Mouse knockout studies are also consistent with the majority of LENK arising from PENK (Gupta et al., 2021). On the other hand, studies supporting PDYN also being a source of LENK include observations that enzymes extracted from the striatum, hippocampus, and substantia nigra metabolize longer dynorphin peptides into LENK (Sandin et al., 1997). The approach used in this study, however, did not limit detection to peptides that are likely released as signaling molecules. Further, destruction of the ventral striatal neurons that project to the substantia nigra that express *Pdyn* and not *Penk* decreased LENK abundance in the nigra (Christensson-Nylander et al., 1986). Together, while evidence is mostly against PDYN being directly upstream of LENK release, it remains plausible that it may occur in specific brain regions or under certain conditions.

Clear functional differences have been observed between dynorphin A and B. Kunselman and colleagues (2021) found that in PC12 cells and cultured striatal neurons, while dynorphin B leads to KOR internalization and then recycling to the plasma membrane, dynorphin A causes internalization with little recovery of receptor to the plasma membrane. They also found that when KOR is activated by dynorphin A, signaling continues after internalization to a much greater extent than when it is activated by dynorphin B. Together these observations indicate a lasting change in receptor signaling and availability that depends upon which dynorphin peptide is released.

Another interesting phenomenon related to PDYN products is that sometimes the cleavage between dynorphin A (1–17) and dynorphin B fails, leaving a much longer functional peptide, big dynorphin (Fig. 2; Merg et al., 2006). Rather than acting through opioid receptors, it appears that big dynorphin acts through glutamatergic NMDA receptors or acid-sensing ion channels type 1a (ASIC1a): using *ex vivo* electrophysiology in acutely dissociated trigeminal neurons, big dynorphin inhibited NMDA receptor mediated currents, and these effects were not blocked by the non-selective opioid receptor antagonist naloxone or the selective KOR antagonist norBNI (Chen et al., 1995a, 1995b). With *in vivo* pharmacology experiments, Kuzmin and colleagues (2006) found that intracerebroventricular injection of big dynorphin increased locomotion and anxiolysis; these effects were not blocked by norBNI, but were blocked by the NMDA receptor antagonist MK-801. They also showed that while dynorphin A and B increased the latency to escape in a hot plate test, big dynorphin did not, strengthening the possibility that there are separate signaling impacts of these peptides (Kuzmin et al., 2006; Tan-No et al., 2002). In cortical neurons, big dynorphin limits the steady-state desensitization of ASIC1a and acid-activated currents, and this effect is blocked by a peptide that binds directly to ASIC1a (Sherwood and Askwith, 2009). More recently it was found that big dynorphin binding to ASIC1a results in a closed resting conformation (Borg et al., 2020). This effect at ASIC1a appears to be mediated by electrostatic interactions of the basic amino acids at the N terminus of big dynorphin, binding in an acidic pocket of the extracellular domain (Borg et al., 2020). The biological conditions that preferentially generate big dynorphin over dynorphin A and B are not yet known.

Another non-opioid receptor mediated effect has also been reported for shorter PDYN fragments. Dynorphin A (1–13) and 2–13 work as antagonists at melanocortin receptors 1, 3, and 4 in the 10s of nM concentration range. However, dynorphin B, nociceptin, β-endorphin, LENK, and MENK did not show this activity (Quillan and Sadée, 1997). An *in vivo* functionality of this interaction has not been identified, but it is provocative in light of the fact that α-melanocyte stimulating hormone (α-MSH), which activates the melanocortin receptors, is one of the peptides encoded by another endogenous opioid peptide precursor, POMC.

### 2.3. Proopiomelanocortin

POMC encodes several opioid and non-opioid peptides, and is the only known precursor for β-endorphin (reviewed in detail in Cawley et al., 2016). It was first isolated from sheep pituitary extract in 1964 (Birk and Li, 1964). POMC is initially cleaved into two peptides, adrenocorticotropic hormone which gives rise to α-MSH, corticotropin-like intermediate lobe peptide, and, at the C-terminal end, β-lipotropin (Fig. 2). β-lipotropin can then be processed to generate the full-length β-endorphin 1–31. β-lipotropin can also produce two other signaling products, γ-lipotropin and β-MSH (Fig. 2). It is not known whether these are necessarily produced during processing to β-endorphin. There are slight differences in the fragments produced in humans compared to rodents (Fricker et al., 2020). While β-endorphin is often thought of as a MOR-favoring peptide, it binds and activates DOR at similar concentrations and KOR with just slightly lower potency (Fig. 1). While β-endorphin 1–31 is often considered to be the signaling peptide, there is evidence that shorter peptides 1–27 and 1–26 may be produced as well. These shorter peptides have recently been shown to have full agonist properties, including having higher efficacy than β-endorphin 1–31 in some signaling assays (Fig. 1; Gomes et al., 2020). As a further consideration, these peptides have been detected both with and without an N-terminal acetyl group, just one of the possible post-translational modifications that are catalyzed at these peptides. This modification prevents binding to opioid receptors (Akil et al., 1984). Interestingly, though MENK is encoded at the address end of the β-endorphins, current understanding is that the pentapeptide is not generated from this precursor.

### 2.4. Pronociceptin

PNOC encodes nociceptin/orphanin-FQ, the endogenous ligand for the opioid-like receptor 1/nociceptin receptor (Houtani et al., 1996; Wang et al., 1996). Interestingly, although there are some sequence similarities between PDYN and PNOC (Houtani et al., 1996), the peptides derived from PNOC have minimal affinity for the other opioid receptors (Fig. 1; Zhang et al., 1997). The propeptide is processed to form 3 primary signaling peptides (Fig. 2): nociceptin/orphanin-FQ, nocistatin (Okuda-Ashitaka et al., 1998), whose target receptor(s) is unclear (Avenali et al., 2016; Kuspiel et al., 2021; Osmakov et al., 2019), and NocII/NocIII that produce behavioral effects when administered intracerebroventricularly (Florin et al., 1997, 1999) but whose effector (s) remains unknown. Although *in vivo* experiments reported that nociceptin and nocistatin might work in opposition (Okuda-Ashitaka et al., 1998), in whole cell electrophysiology in locus coeruleus (LC) neurons nocistatin application did not activate a conductance and it also did not block responses to nociceptin (Connor et al., 1999).

### 2.5. Endomorphins

The tetrapeptides endomorphin-1 (Tyr-Pro-Trp-Phe-NH_2_, YPWF, EM-1) and endomorphin-2 (Tyr-Pro-Phe-Phe-NH_2_, YPFF, EM-2) are unique among the endogenous opioid peptides that bind to the MOR in that they are quite selective for this receptor (Fig. 1; McConalogue et al., 1999; Zadina et al., 1997), however their synthesis pathway remains unclear and their classification as “endogenous” has been in question over the years. The EMs were originally detected in brain and spinal cord principally with radioimmunoassays (RIAs) using antibodies raised against synthetic peptide antisera (Narita et al., 1998; Xie et al., 2008; Zadina et al., 1997). It is important to note that RIA detection of small peptides such as EM-1 and EM-2 can lack selectivity, and without EM knockout tissue to validate antibody selectivity some questions have remained in the field. Alternatively, the chemical identity of detected peptides can be definitively characterized through mass spectrometry or Edman degradation, techniques to sequence short peptides. Accurate detection methods are particularly critical for EMs because conventional precursor mRNA sources for the EMs have not been found. Three bioinformatic publications identify potential endogenous sources for EM-1 and EM-2, which we describe in detail.

First, Jessop and colleagues (Jessop et al., 2000), searched uniprot 21 for YPWFG as Gly is required for C-terminal amidation (Eipper et al., 1992), however this only gave hits in honeybee, dogfish, or influenza virus, while YPFFG only gave two hits in plants.

Second, Terskiy et al. (2007) expanded the search to known and predicted human sequences in NCBI RefSeq (9/2006), SwissProt (10/2006), and TrEMBL (10/2006) for EM-1 and EM-2 sequences with and without the terminal Gly and with and without a terminal basic residue, further extending the recognition motif for C-terminal amidation. They similarly found hits for sequences containing C-terminal Gly motifs, SUT-1 sodium/sulfate symporter (SLC13A4), and coiled-coil domain containing 100 (CCDC8). The expression pattern for these, however, do not match EM-1 or EM-2 RAI staining, as SLC13A4 is predominantly expressed in placenta and testis with intermediate levels in brain and lower levels in heart, thymus, and liver (Girard et al., 1999), and CCDC8 is widely expressed with low levels in spleen, skeletal muscle, small intestine, kidney, and liver (Hanson et al., 2011). They also found hits without any C-terminal amidation motif, which are likely not biologically relevant. For EM-1, the 12 hits included SH2 containing proteins, olfactory receptor 51V1, trace amine-associated GPCRs 1–5 and Gα_q_s, which are effectors of MOR among other GPCRs (Barreto et al., 2021; Minami et al., 2010; Psifogeorgou et al., 2011). For EM-2, the 20 the hits were more diverse, including Galacto-cerebrosidase, Ataxin 1, choline kinase, Insulin-like growth factor receptor 1 precursor, and secreted frizzled-related protein 5. Hanson et al. (2011) showed by size exclusion that EM-2 immunostaining can stain multiple fractions of rat brains ranging in size from 25 to 117, suggesting they may stain one or more of these proteins (Terskiy et al., 2007).

While most opioid ligands are cleaved, it is possible that the YPWF and YPFF sequences are not. Longer proteins with these sequences might bind and exert effects at MOR, either in an attached loop, attached at a single end, or free at both ends without the C-terminal amidation. Building on Schwyzer’s message-address concept (Schwyzer, 1977) for opioid receptors (Portoghese, 1989), and extensive structure activity relationship development and structural modeling (Pasternak and Pan, 2013; Yu et al., 2007), it is widely assumed that the N-terminal tyrosine buries deep into the GPCR extracellular facing active site, with the C-terminus pointing outwards. Thus it is unlikely that EM-1 could be recognized with N-terminal extensions. Since the C-terminus is pointing outwards, it is possible that it is attached to a longer polypeptide chain, as it is for example in endorphins. If there is no C-terminal extension, then C-terminal amidation has long been recognized as important for MOR selectivity (Rónai et al., 1979) and indeed, EM-1OH and EM-2OH are reported to be either 2–4x less potent (Al-Khrasani et al., 2001) or 200–300x less potent (In et al., 2005) than EM-1 or EM-2.

Third, Matsushima, Sese, and Koyanagi (2019) further expanded the bioinformatic EM search to the mammalian expressed sequence tag database (Boguski et al., 1993), containing reads from shotgun mRNA sequencing deposited into NCBI prior to assembling into transcriptome profiles. They obtained one hit RYP**W**FGR in AI352151.1, however the position in human chromosome 8 differs RYP**G**FGR, and they confirmed this substitution with cDNA cloning. While Matsushima and colleagues showed that YP**G**F-NH_2_ has no appreciable activity at MOR, they noticed that the codon for G (GGG) only differs from that of W (TGG) by a single G->T base substitution and that the relatively common oxidation of guanine at position 8 (8-oxoguanine) may cause G->T substitution (Kanvah et al., 2010). Further, triple oxidation GGG -> TTT would generate the F needed for EM-2. It is unclear whether the required high levels of oxidative stress would occur *in vivo*. Acute hard exercise or chronic Fibromyalgia generate oxidative stress and have been associated with pain and reward, potentially due to EM release (Cordero, 2011; Hendrix et al., 2020). However, highly oxidative conditions may be particularly exaggerated in certain *ex vivo* tissue preparations (Ravanat et al., 2002). Using BLASTn to search for the cDNA nucleotide sequence among human transcripts, Matushima and colleagues found a strong match that spans adjacent DDHD domain-containing protein 2 (DDHD2) and *Pan troglodytes* nuclear receptor binding SET domain protein 3 (NSD3) predicted genes (2019).

To complement the characterization of potential source(s) of EM-1 and EM-2, a crucial dimension of its endogenous regulation is understanding how it may be degraded. EM-1 and EM-2 are substrates for the broadly expressed serine proteinase dipeptidyl peptidase IV (DPP4), which removes xP- from amino terminal peptides (Sakurada et al., 2003; Shane et al., 1999). DPP4 is well studied for its degrading glucagon-like peptide 1 (GLP-1) and the treatment of type-2 diabetes (Demuth et al., 2005), as well as other signaling peptides including substance P (Mentlein, 1999), and there are several FDA approved drugs targeting DPP4. Modulation through DPP4 is an interesting strategy to probe EM function as a potential therapeutic strategy to treat pain. For example, Shane et al. (1999) showed that Ala-Pyrrolidonyl-2-nitrile (ala-Pyrr-2-CN) inhibits DPP IV competitively with a K_i_ of 0.2 μM, modulated EM-2 effects on the tail-flick pain assay. A range of experiments using DPP4 inhibitors and exogenous EM-2 in animal models suggest a link. For example, by using a DPP4 knockout mouse model, Komiya et al. (2022) showed that while DPP4 inhibition of substance P metabolism increases psoriatic itch, EMs drove mechanical allokinesis through expression in the squamous and basal layer keratinocytes of the epidermis. Beyond DPP4, the less studied DPP3 has also been shown to metabolize EMs at similar enzymatic efficiency (Baršun et al., 2007), though the *in vivo* function is less well characterized (Malovan et al., 2022).

While definitive characterization of EM-1 and EM-2 production and degradation remains to be clarified, these studies together constrain the hypothesis space. Given the high-potency and selectivity of EM-1 and EM-2 for MOR, the robust detection of EM-1 and EM-2 with RIA, and modulation of MOR signaling by DPP4 suggests they are biologically produced and form an important dimension of endogenous MOR signaling. Therefore, a deeper understanding potential regulation of these processes may open up new therapeutic strategies and liabilities for treating pain and substance abuse disorder.

## 3. Endogenous opioid peptides are expressed in specific neural circuits

### 3.1. Introduction to peptide distribution studies

Opioid peptide and receptor distributions in the central nervous system (CNS) have attracted intense research efforts to identify the neural circuits that potentially contribute to both their beneficial and deleterious effects. Of note, opioid receptors are expressed in several non-neuronal organs including heart and immune cells as well. The highest concentrations of endogenous opioid peptides are found in the adrenal gland, where they are synthesized by adrenal medullary chromaffin cells that release the opioid peptides into the adrenal vein for systemic circulation (Livett et al., 1981; Viveros et al., 1979). In brain, early on the highest concentration of MENK was found in the globus pallidus (Kobayashi et al., 1978). Intense ENK immunoreactivity was detected in the periaqueductal gray (PAG), a key brain region associated with pain transmission and opioid analgesia (Moss et al., 1983; Moss and Basbaum, 1983). In many brain regions ENK and dynorphin expression patterns have been found to be complementary, most easily discerned through mRNA detection (See Section 3.4).

Much research has also focused on how levels of opioid precursor mRNA or peptide content in different brain regions change following a wide variety of *in vivo* experiences. The functional implications for peptide release of changes in tissue peptide content or mRNA levels, however, remain mostly unknown, as changes in mRNA levels do not necessarily correlate with changes in peptide expression levels (e.g., Greenbaum et al., 2003; Guo et al., 2008; Haque et al., 2017; Tang et al., 2009). Extracellular measurements to detect exocytosed peptide are critical for the field’s understanding, and more temporally precise methods are required to align observed peptide release with specific behavioral events (Abraham et al., 2021; Calhoun et al., 2018; Conway et al., 2022; DiFeliceantonio et al., 2012; Girven et al., 2022; Mabrouk et al., 2011).

More recently immunocytochemical labeling has revealed distinct expression patterns for EM-1 and EM-2. EM-1 is preferentially detected in brain in the parabrachial nucleus, the nucleus of the solitary tract (NTS), septum, diagonal band, bed nucleus of the stria terminalis (BNST), hypothalamus, PAG, nucleus accumbens (NAc), LC, and amygdala (summarized in Fichna et al., 2007; Martin-Schild et al., 1999, 1997). In contrast, EM-2 is preferentially expressed in the spinal cord. Importantly, chemically stimulated release of EM-2 was detected in the spinal cord using HPLC and electrochemical detection in samples collected with push-pull perfusion (Lisi and Sluka, 2006).

### 3.2. Regions of endogenous opioid peptide mRNA expression

Mappings of brain regions with high expression levels of endogenous opioid peptide precursor genes have been reviewed elsewhere, including patterns of changes in expression levels following different *in vivo* treatments (Merrer et al., 2009). Briefly, high expression levels of endogenous opioid peptide mRNA is detected in many neurons in the hypothalamus, striatum, pallidum, amygdala, extended amygdala, PAG, spinal cord, and dorsal root ganglia, especially for *Penk* and *Pdyn* (e.g., Fallon and Leslie, 1986; Manzanares et al., 1998; Neal and Newman, 1989; Olsen et al., 2008; Sapio et al., 2020; Schlussman et al., 2011; Solecki et al., 2009; Sukhov et al., 1995). The main populations of POMC expressing neurons reside in the arcuate nucleus of the hypothalamus and the commissural NTS. POMC is also expressed in cells of the anterior pituitary (Bloch et al., 1979; Bloom et al., 1978; Jacobowitz and O’Donohue, 1978; Sofroniew, 1979). Recently, mice have been engineered to express Cre recombinase under the peptide precursor genes, which enable more precise identification of the neurons that express the opioid peptide precursor mRNA and facilitate mapping of their neural circuits (e.g., Viden et al., 2022).

PNOC is expressed widely throughout the brain, overlapping with many regions that express other endogenous opioid peptides. Mice that express Cre recombinase under the *Pnoc* promoter, enabling identification and manipulation of these neurons, have been used to investigate the distribution of *Pnoc* expressing neurons (Hardaway et al., 2019). In these animals *Pnoc* expressing neurons have been studied in the central nucleus of the amygdala (CEA), BNST (Rodriguez-Romaguera et al., 2020), and in a subregion of the ventral tegmental area (VTA) adjacent to the interpeduncular nucleus (Parker et al., 2019).

The peptide distribution observations made using *in situ* hybridization of mRNA identify regions with relatively high expression. While these regions are a logical focus, it is also possible that important populations of neurons that express endogenous opioid peptides fall below detection limits or analysis thresholds in these studies. Further, neuropeptides are generally released from axon terminals, often projections to distant brain regions, therefore techniques that identify somata or nuclei that express mRNA for precursors do not reveal where the opioid peptides are released (see Section 4 below). That said, exciting advances in mRNA detection have enabled new, detailed studies of expression patterns and how they change with behavioral state, contributing to our understanding of how neural circuits change with *in vivo* experiences.

### 3.3. New possibilities with single cell and low abundance mRNA detection: case study in the VTA

Genetic sequencing technology has advanced significantly such that high-resolution, high-throughput detection is now possible, including identifying low abundance signals at the single cell level (Svensson et al., 2017). With appropriate controls, there is no practical limit to how many transcripts can be detected in each cell via RNAseq, providing a different, and potentially very exciting, kind of information compared to techniques that require higher abundance of target material for detection. By isolating a brain region, one can then determine the transcripted mRNAs from each isolated cell including neurons, glia, and epithelial cells. These techniques require careful gross dissection and isolation of the desired brain region. Some structures naturally lend themselves to accurate isolation, but others do not. This can make it challenging when attempting to interpret low-expression mRNAs or low-abundance clusters, as they may either represent small populations in the target brain region or contamination from neighboring brain regions. For higher abundance mRNAs or proteins, distributions can be subsequently confirmed with RNAscope or immunofluorescence. Spatial transcriptomics quality is also rapidly improving to provide topographical organization information (Beauchamp et al., 2022). When used appropriately, these techniques can be incredibly powerful, both for confirming earlier observations and for detecting previously unknown expression patterns, thereby enabling the generation of novel hypotheses.

For example, unbiased populations of VTA cells from male and female rats were recently profiled with single nucleus RNA sequencing by Phillips and colleagues (2022). In their analysis of the dataset they focused on the expression of opioid peptide related genes. With respect to the conventional neurotransmitters associated with VTA neurons, dopamine, glutamate, and GABA, the combinatorial expression patterns that were previously reported (reviewed in Morales and Margolis, 2017; Barker et al., 2016) were also detected in this study. That is, there is anatomical, physiological, immunocytochemical, and conventional *in situ* hybridization evidence for VTA neurons that have the capacity to synthesize and signal through more than one neurotransmitter including dopamine/glutamate, glutamate/GABA, and dopamine/glutamate/GABA cell populations in the VTA. These different types of VTA neurons participate in specific neural circuits (Morales and Margolis, 2017). One feature of this RNAseq dataset is that it expands the profiling of the genes that contribute to the synthesis, transport, and degradation of these transmitters, all of which provide additional evidence that neurons can in fact synthesize and release each neurotransmitter. For example, they examine several genes involved in the synthesis of tetrahydrobiopterin, which is essential in the first step of dopamine synthesis, generating L-DOPA from tyrosine (Phillips et al., 2022). With respect to genes underlying endogenous opioid peptide production and the opioid receptors, their clustering shows that *Penk* is relatively highly expressed in the VTA glutamate-only neuron subcluster. *Penk* expression was also detected in small subpopulations of glutamate/GABA neurons, and a subcluster of GABA only neurons. Message for *Pomc* or *Pdyn* was detected just sparsely in VTA neurons, consistent with prior studies. *Pnoc* was detected among several cluster phenotypes of GABA only neurons and one cluster of glutamate/GABA neurons, consistent with transgenic mouse results (Parker et al., 2019). It is possible that these patterns relate to the projection targets of the VTA neurons, as has been reported for clusters of gene expression detected when dopamine neurons have been studied in isolation (Poulin et al., 2018). Evaluating the expression of the enzymes required for synthesis of the signaling peptides from the propeptides will help determine whether these newly resolved populations of VTA neurons can indeed generate the endogenous opioid peptides. All four opioid receptors were detected in this VTA study, with *Oprm1* being most common and detected in many neurophenotypic clusters, followed by *Oprd1*, with much lower detection frequencies of *Oprk1* and *Oprl1*. These findings are in contrast to strong detection of *Oprk1* expression in dopamine neurons using RNAscope (Liu et al., 2019) and KOR activity using physiological techniques (Ford et al., 2007; Margolis et al., 2003, reviewed in Margolis and Karkhanis, 2019), as well as the physiological responses to nociceptin reported in both dopamine and non-dopamine VTA neurons (Driscoll et al., 2020; Zheng et al., 2002). Together, this kind of analysis of endogenous opioid peptide precursor message as well as that for their receptors can provide new perspectives on expression patterns, but need to be interpreted in the context provided by other techniques.

### 3.4. Patterns of opioid peptide mRNAs in specific neural circuits

Importantly, peptide-releasing neurons participate in specific neural circuits. In several brain regions it is established that the particular endogenous opioid peptides synthesized by a subpopulation of neurons has a strong correlation with the projection target of that subpopulation. One clear example of this is the organization of the *Penk-* and *Pdyn*-expressing neurons in the dorsal striatum and NAc (ventral striatum). Here, projection neurons are medium spiny (MSN) in morphology, release GABA as a fast neurotransmitter, and express several neuropeptides. The “indirect” pathway neurons of the dorsal/ventral striatum send axons to the globus pallidus external/ventral pallidum but not the substantia nigra/VTA; these neurons express *Penk* and dopamine D2 receptors (*Drd2*; Gerfen et al., 1990). The “direct” pathway neurons send axons to the substantia nigra/VTA and express *Pdyn* and protachykinin (precursor to substance P; Anderson and Reiner, 1990; Gerfen and Young, 1988), as well as dopamine D1 receptors (*Drd1*; Gerfen et al., 1990; Vincent et al., 1982). In the NAc, *Drd1* MSNs also project to the ventral pallidum, thus do not exclusively project “directly” to the VTA (Kupchik et al., 2015). For a detailed review of the organization of the NAc with a particular focus on neuropeptides we recommend (Castro and Bruchas, 2019).

In other subregions of the extended amygdala there is also clear organization of *Penk* and *Pdyn* neurons. For instance, there is a substantial population of *Penk* neurons that are glutamatergic (Poulin et al., 2008). In the CEA, PDYN is expressed in a subset of neurons, many of which also express corticotropin releasing factor (Marchant et al., 2007). Indirect evidence is consistent with PENK being expressed in a non-overlapping subpopulation of CEA neurons (Marchant et al., 2007). CEA *Penk* neurons innervate the basolateral amygdala (BLA), BNST, substantia innominata, ventral pallidum, many regions in the hypothalamus, pedunculopontine tegmental nucleus, retrorubral field, substantia nigra, VTA, NTS, and trigeminal nucleus (Viden et al., 2022). In the BNST, *Pdyn* is expressed specifically in the fusiform, oval and anterior lateral nuclei (Marchant et al., 2007; Poulin et al., 2009). Unlike in the CEA, corticotropin releasing factor is not expressed in *Pdyn* neurons in the BNST, thus coexpression of different peptides varies across brain regions (Marchant et al., 2007). *Penk* expression is more widespread in the BNST, but low or no expression was detected in the fusiform and rhomboid nuclei (Poulin et al., 2009). In the anterior BNST, all *Penk* neurons were GABAergic (Poulin et al., 2009), but in the posterior BNST a small percentage of *Penk* neurons were found to be glutamatergic, not GABAergic (Poulin et al., 2009). The CEA and BNST share reciprocal *Penk* connections (Viden et al., 2022). Another example of a brain region with separate PENK and PDYN cells is the NTS (Lee and Basbaum, 1984). Thus, even though *Penk* and *Pdyn* are often expressed in the same brain region, they are consistently expressed in different neural subpopulations.

Arcuate nucleus *Pomc* neurons project widely, including targeting the BNST, lateral septal nucleus, NAc, PAG, supraoptic nucleus, VTA, dorsal medial hypothalamus (DMH), paraventricular nucleus of the hypothalamus (PVH), lateral hypothalamus, periventricular nucleus, amygdala, dorsal vagal complex, and NTS (King and Hentges, 2011; Metz et al., 2021, reviewed in Cone, 2005). Recent work demonstrates that there are two populations of *Pomc* neurons in the arcuate nucleus, distinguished by their expression of either the leptin receptor or the glucagon-like peptide 1 receptor (Biglari et al., 2021). Both of these populations contribute to major *Pomc* projections including to the BNST, DMH, PAG, PVH, and NTS (Biglari et al., 2021). In spite of this, for the most part arcuate POMC axons do not collateralize to different brain regions (Metz et al., 2021). *Pomc* neurons in the NTS innervate the caudal mesencephalon and spinal cord. LC, parabrachial nucleus, rostral NTS, dorsal motor nucleus of the vagus and lateral reticular nucleus receive *Pomc* axons from both the arcuate and the NTS (reviewed in Cone, 2005). Together, these relatively small populations of *Pomc* neurons innervate a great deal of the CNS.

Studies like these that demonstrate patterns of expression and are very powerful for improving our understanding of the possible range of neurotransmitters and neuromodulators that may be used for communication between neurons in specific circuits. Yet they do not help us resolve where or when the specific neuromodulators are released. Neurons have highly specialized subcellular compartments and many molecules are trafficked to specific parts of the neuron, even to specific subsets of axon terminals or neurotransmitter release sites within a single axon (Scanziani et al., 1998; Silm et al., 2019; Zhang et al., 2015). Therefore it cannot be assumed, for instance, that a neuron that expresses *Penk* releases enkephalins at all of its synaptic sites or in local dendritic compartments.

## 4. Subcellular compartmental distribution of opioid peptides

**Fig. 3.**
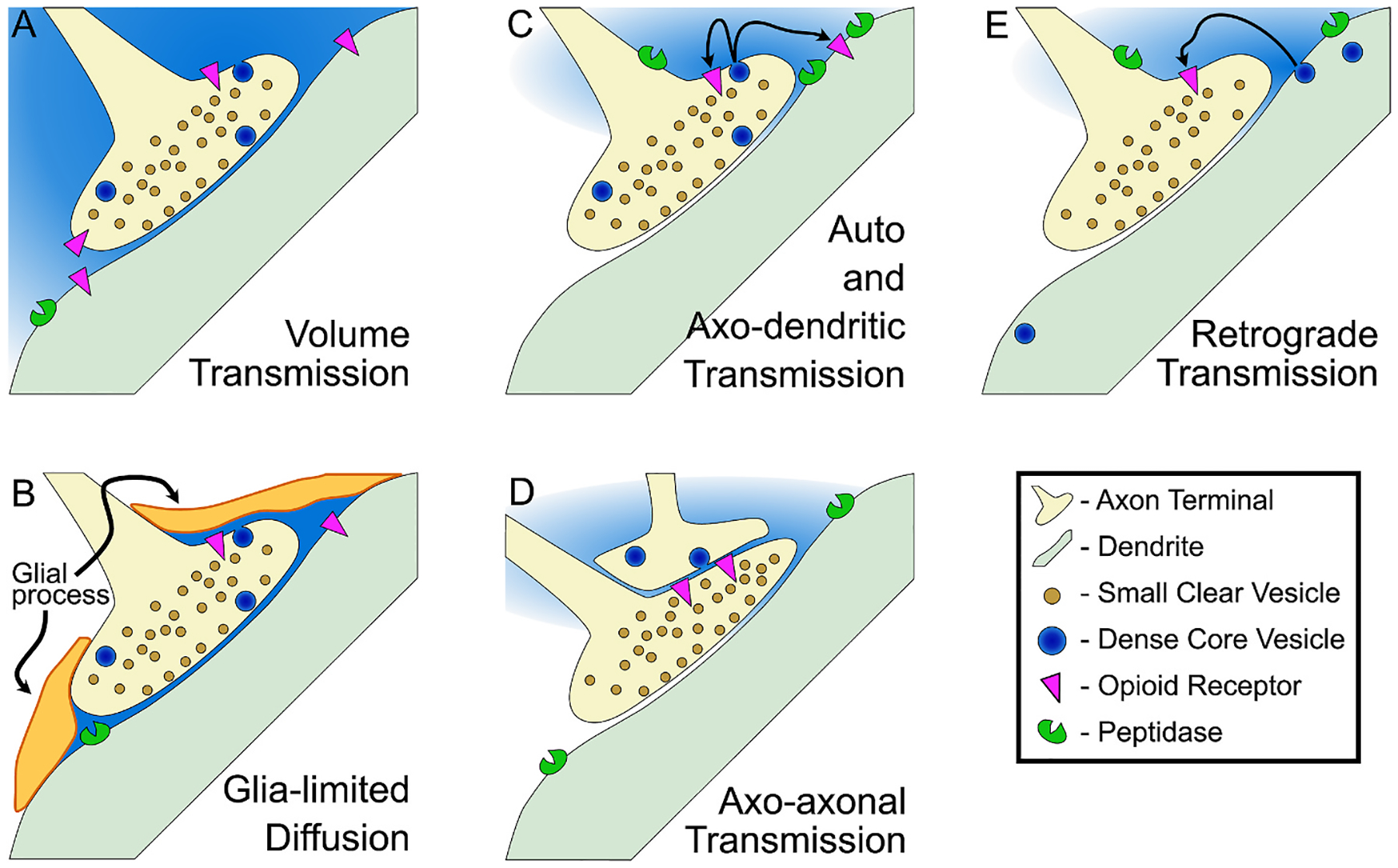
Arrangements of endogenous opioid peptide neurotransmission. (A) The features of the volume transmission model are that endogenous opioid peptides diffuse relatively freely from the release site, reaching opioid receptors that are 10s–100s of μm away from the release site, and acting on a slow time course, modulating neural activity on the scale of minutes or longer. Several biological mechanisms that oppose the volume transmission model have been observed. (B) In some brain regions glial processes seem to wrap axon terminals that have endogenous opioid-containing dense core vesicles, potentially limiting peptide diffusion. (C) Opioid peptide-containing vesicles have mostly been detected in axon terminals and varicosities, from which they may signal to opioid receptors on the same axon or on a nearby dendrite. (D) In some brain regions opioid-containing vesicles were found in axons that appose another axon terminal, and often these terminals express opioid receptors. (E) In a few cases endogenous opioid peptide-containing vesicles have been detected in dendrites, and it is postulated that the peptides released from these dendrites may signal retrogradely to opioid receptors on nearby axon terminals.

It is generally thought that propeptides are processed into signaling neuropeptides in rough endoplasmic reticulum then transported through the Golgi apparatus to be packaged into the dense core vesicles from which the peptides are released to the extracellular space. Large dense core vesicles are 80–120 nm in diameter, compared to small clear vesicles that contain fast neurotransmitters and are on average 40 nm in diameter. Therefore putative peptide-containing vesicles can be visually identified by size with sufficient magnification. In ultrastructure studies, dense core vesicles containing endogenous opioid peptides have mostly been observed in axon varicosities and axon terminals, with much less localization to dendrites and rare localization in somata. These vesicles are usually present in low abundance in any of these compartments. Terminals are typically filled with tens to hundreds of small clear vesicles but often co-contain no more than 2–3 dense core vesicles, for example reported in dynorphin containing terminals (Pickel et al., 1993) and ENK containing terminals (Sesack and Pickel, 1992) in the VTA. The apparent scarcity of dense core vesicles is curious given that it is generally thought that neuropeptides signal through volume transmission (Fig. 3A), diffusing far from release sites. This would probably require multiple release events in order to achieve pharmacologically relevant extracellular concentrations over a large volume of tissue.

**Fig. 4.**
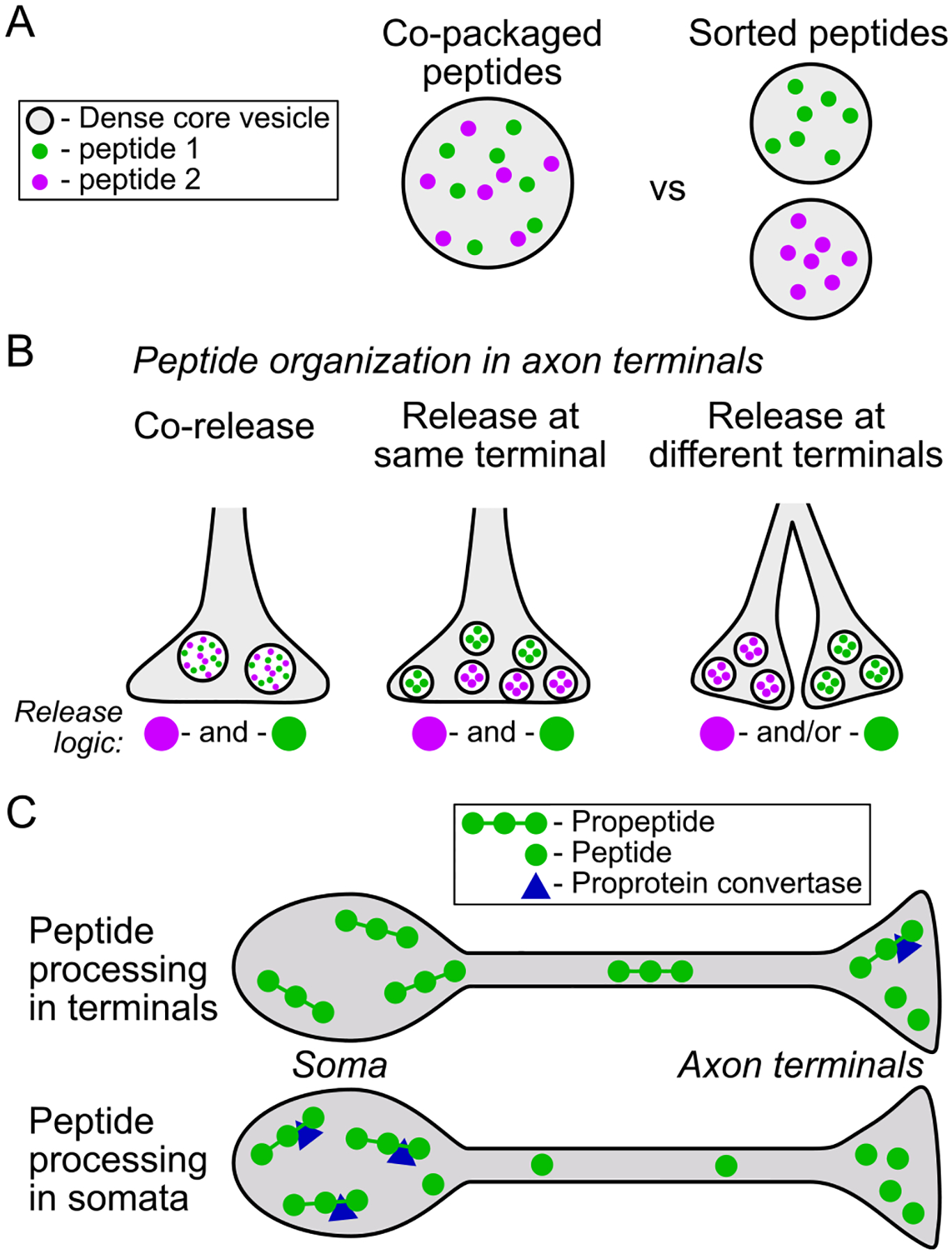
Neurons may sort peptides for co-release or independent release. (A) In different brain regions, neuromodulator peptides have been found to be packaged into the same vesicle resulting in simultaneous release or in separate vesicles potentially enabling independent release. (B) At the level of release sites, there is evidence that in some systems different signaling molecules, including different peptides, are segregated to different release sites. Various physiological phenomena may then independently control release events at the different synaptic boutons in the right hand case. (C, top) propeptides may be synthesized in the soma or axon terminals, and are mostly cleaved to shorter signaling molecules in axon terminals. This arrangement enables rapid changes in peptide production at the level of axon terminals. (C, bottom) in other neurons, propeptides appear to be delivered to axon terminals already processed and released without further modification.

Many types of neurons express neuropeptides that are generally thought to have opposing physiological effects, such as lateral hypothalamic neurons that express both orexin (hypocretin) and PDYN (Chou et al., 2001), or striatal MSNs that express substance P and PDYN (Anderson and Reiner, 1990; Reiner, 1986). In a simple model where excitatory and inhibitory signals compete to control neural activity, such expression patterns are hard to interpret. One possibility is that the critical downstream intracellular signaling effects of the neuropeptides are as important, if not more important, than the modulation of contemporaneous action potential activity. Another is that the time course of the receptor responses differ. If something like these possibilities is the case, segregated vesicular loading would seem unnecessary (Fig. 4A). Sorting of peptides into different vesicles may also be unnecessary if neuromodulators signal through volume transmission (Fig. 3A; Agnati et al., 1995). Another possibility is that peptide transmission is more targeted, that the wiring diagram for neuropeptide control of neural circuits has higher spatial resolution than volume transmission (Fig. 3B–E). There is evidence that both of these arrangements are present in the brain.

There are several reports of detection of colocalization of different peptides in the same dense core vesicles (Fig. 4A and B). For opioid peptides, one example is in lateral hypothalamus neurons where the generally excitatory neuropeptide orexin is colocalized with dynorphin in dense core vesicles found in axon varicosities, axon terminals, and dendrites (Muschamp et al., 2014).

On the other hand, in neurons of the arcuate nucleus of the hypothalamus, neuropeptides generated from different precursor molecules are not co-packaged into the same dense core vesicles (Fig. 4A and B). In neurons that produce kisspeptin, neurokinin B, and dynorphin, neither kisspeptin nor neurokinin B was found to colocalize with dynorphin A in dense core vesicles in the somata or axons of these neurons (Murakawa et al., 2016).

The peptides synthesized in POMC neurons, including different peptides that are cleaved from POMC itself, may be aggregated at specific release sites (Fig. 4B). For instance, in the dorsal raphe nucleus (DRN) most axon terminals that are labeled for β-endorphin are in the dorsomedial DRN. These axon terminals tend to contain either a mix of dense core vesicles with small clear vesicles or mostly dense core vesicles, often synapsing onto dendritic spines of GABAergic profiles (Wang et al., 2001). However, in a separate study it was reported that axon terminals labeled for adrenocorticotropic hormone, also produced from POMC, contain an abundance of small clear vesicles with few dense core vesicles synapsing mostly onto dendritic shafts (Léger et al., 1997). While this is not direct evidence against colocalization, the differing arrangements are consistent with specific axon terminals releasing different peptides (Fig. 4). As proof-of-principle, separate packaging of the different peptides generated from the egg-laying hormone propeptide in Aplysia has been demonstrated to occur through sorting at the trans-Golgi (Fisher et al., 1988).

The extent of processing of propeptides prior to packaging into dense core vesicles and transporting of these vesicles to release sites may vary by neuron type. In some neurons there is evidence that propeptides are processed in axon terminals (Riesenberg and Nitsch, 1990), while in other neurons high concentrations of signaling peptides like MENK have been detected in somata (Fig. 4CB; e.g., Pickel et al., 1980). The processing enzymes prohormone convertases PC1 and PC2 have been detected at high levels in axon terminals throughout the brain (Winsky-Sommerer et al., 2000), suggesting that much propeptide processing occurs at terminals just prior to release. This has been investigated in detail in the VTA, where *Pdyn* mRNA has not been detected in somata. Therefore if present, both the longer and shorter peptides almost definitely reside in innervating axons. Using immuno-electron microscopy, both PDYN and dynorphin A were detected in axon terminals in the VTA (Yakovleva et al., 2006). Further, using western blotting, the concentration of PDYN was found to be much higher than dynorphin in VTA tissue samples (Yakovleva et al., 2006). C-terminal processing intermediates were also detected in the VTA (Yakovleva et al., 2006). This is consistent with much of the processing of PDYN into dynorphin A occurring in the axon terminals in the VTA, rather in the cell body region from which these axons originate such as the MSNs in the NAc (Yakovleva et al., 2006). Such an arrangement enables more rapid adaptation of peptide production and release than when peptide production is completed in somata and requires axonal transport to release sites.

Alternatively, there is evidence that EM-2 may be produced and packaged in the somatodendritic region of hypothalamic neurons before being trafficked axon terminals (Fig. 4C). By electron microscopy, EM-2 was detected in dense core vesicles in somatodendritic regions in the dorsomedial and ventromedial hypothalamic nuclei and the region near the third ventricle (Wang et al., 2003). EM-2 immunoreactivity was detected in granules in the Golgi apparatus, which likely give rise to EM-2-containing dense core vesicles (Wang et al., 2003). EM-2-labeled dense core vesicles were also detected in the axon terminals in these regions, therefore these dense core vesicles may be trafficked to these local terminals for release (Wang et al., 2003). The synaptic specializations where EM-2 dense core vesicles were detected contained mostly small clear vesicles and were predominantly asymmetric, consistent with excitatory glutamatergic type synapses (Wang et al., 2003). This difference in synthesis and packaging location may be related to the substantial differences in peptide generation for the endomorphins compared to the other endogenous opioid peptides.

## 5. Peptide release and volume of influence

In electron microscopy studies where synaptic appositions have been identified, most dense core vesicles are located somewhat distal from the synaptic specialization or active zone, the primary release site for small clear vesicles (e.g., Sesack and Pickel, 1992; Zhu et al., 1986). This observation has led to the proposal that dense core vesicles generally release their contents extrasynaptically (Fig. 3). One caveat to this idea is that in studies where samples were preserved with rapid freezing techniques indicate chemical fixation leads to some distortions of pre-synapse organization, including vesicle tethering and distributions (Bruckner et al., 2015). Fusion events of dense core vesicles can depend on some membrane proteins that are shared with small clear vesicles, such as the synaptosome-associated protein receptor (SNARE) proteins vesicle-associated membrane protein (VAMP), syntaxin, and synaptosome-associated protein [~25 kDa] (SNAP-25; Bruckner et al., 2015; Gormal and Meunier, 2022), but some are different such as calcium-dependent activator protein for secretion (CAPS; Berwin et al., 1998; Farina et al., 2015; Fujima et al., 2021; Kasai et al., 2012). Depending on the complement of fusion related proteins expressed on the plasma membrane of a population of dense core vesicles, different release sites for neuropeptides separate from the synaptic specialization may be enabled or required.

However, there is also some evidence that dense core vesicles do release their contents directly into synaptic specializations. For instance, dense core vesicle fusion directly in the presynapse at the neuromuscular junction has been detected (Jung et al., 2018). Where peptide release generally involves calcium dependent fusion machinery, in order to be exposed to adequate calcium concentrations to drive release the dense core vesicles likely need to be within tens of nm of calcium channels (Schneggenburger et al., 2012; Wang and Augustine, 2015). It is worth noting that calcium channels aggregate in the active zone, more so than in adjacent membrane, therefore dense core vesicles that require high concentrations of calcium for release may fuse in the active zone of the synaptic contact (Kaeser et al., 2011; Kusch et al., 2018).

A major question is what is the volume of tissue whose function is impacted by endogenous opioid peptide release from a given site. The dominant model is that neuromodulators, including peptides, act through volume transmission (Fig. 3A). However, some studies lend support to a model where neuropeptide diffusion, including of endogenous opioid peptides, is limited by several factors. For instance, it is not unusual for glial processes to be detected covering the extrasynaptic areas that are inferred to be opioid peptide release sites in ultrastructure studies, such as for MENK terminals in the LC (Bockstaele et al., 1995) and VTA (Sesack and Pickel, 1992). Similar astrocytic processes were reported in the LC near putative release sites for β-endorphin (Bockstaele et al., 1995; Reyes et al., 2006), in the VTA near putative release sites for LENK (Sesack and Pickel, 1992, 1995), and in the substantia nigra pars reticulata near putative release sites for dynorphin (Pickel et al., 1995). It is possible that the function of this astrocytic arrangement is parallel to the regulation they provide of fast neurotransmitter synaptic connections, akin to a peptide active zone, guiding or limiting the diffusion of the peptides towards structures that contain the relevant receptors (Fig. 3B).

Enzymatic breakdown of extracellular endogenous opioid peptides is also quite rapid and likely limits diffusion (Fig. 3C–E). Aminopeptidase B and endopeptidase-24.11 (metalloendopeptidase/neprilysin) are membrane bound enzymes (Larrinaga et al., 2011; Mumford et al., 1981). The localization of these peptidases to specific neural and cellular compartments is not broadly established. Distributions of two endopeptidases were studied pig substantia nigra, endopeptidase-24.11 and angiotensin converting enzyme (Barnes et al., 1992). Endopeptidase-24.11 was detected in a subset of dendrites, and in axons was localized specifically to boutons, with a tendency for labeling to be more intense at or adjacent to synaptic contacts. Although measures were indirect, this study further concluded that angiotensin converting enzyme was more likely to be expressed on postsynaptic membranes rather than on axons. Axons, dendrites, and glial processes contained endopeptidase-24.11 in the NTS (Barnes et al., 1993; Lasher et al., 1990). Bouton expression of endopeptidase-24.11 was observed in the globus pallidus, localized to axonal plasma membranes as well as synaptic boutons, but not dendrites or glial membranes with electron microscopy (Barnes et al., 1988b). Expression levels of these enzymes vary markedly across brain regions; for instance there is very low abundance of angiotensin converting enzyme in globus pallidus and olfactory tubercle (Barnes et al., 1988a). We discuss how blocking opioid peptide catabolism enables the detection of endogenous opioid peptide release effects *ex vivo* below (Section 7), consistent with a strong regulatory role of these enzymes to limit peptide diffusion.

## 6. Receptor proximity to peptide release sites

Terminals that contain endogenous opioid peptides have mostly been detected to synapse onto dendrites and other axon terminals in ultrastructural studies (Fig. 3C and D). Far less frequently axon terminals containing opioid peptides have been detected synapsing onto somata, such as dynorphin A terminals synapsing onto cell bodies in the substantia nigra pars compacta (Riesenberg and Nitsch, 1990). An important question is: how close are opioid receptors to the release sites of endogenous opioid peptides? Similar to the ultrastructural localization of opioid peptide containing dense core vesicles, opioid receptors are also generally localized extrasynaptically, near the expected release sites for opioid peptide containing dense core vesicles (e.g., Aicher et al., 2003; Bockstaele et al., 1996, 1997). Are opioid receptors *more likely* to be near peptide release sites compared to more distant neuronal profiles?

There is some evidence that subpopulations of GPCRs are anchored to specific locations on the plasma membrane (Thurner et al., 2014; Yanagawa et al., 2018) including MORs (Metz et al., 2019; Suzuki et al., 2005). These single receptor tracking techniques suggest that individual receptors move within plasma membrane areas on the order of <10 μm^2^, consistent with some compartmentalization. Another clue for targeted localization of GPCRs to specific neural compartments is the distribution of G proteins. A recent study reported the distribution of Gαo proteins in Purkinje neurons in mouse cerebellum (Roldán-Sastre et al., 2021). Using immunocytochemical labeling of the G protein and localization with electron microscopy, Roldán-Sastre and colleagues reported that for Purkinje neurons most labeling was within dendritic spines, virtually no labeling was localized to somata, with low labeling in main/proximal dendrites and moderate labeling in spiny branchlet dendrites. Further, frequency of Gαo particle detection peaked at 180 nm from the synaptic specialization, with much lower density reported at distances ≥300 nm. Gαo particles were also located within the postsynaptic density, closer to the peripheral edges than to the center (see also Fernández et al., 2009). It is unknown whether these might be associated with neuropeptide receptors like opioid receptors or GPCRs that respond to fast neurotransmitters such as GABA_B_ receptors or metabotropic glutamate receptors. While we do not know from this report the distribution of specific GPCRs relative to the Gαo, it does suggest some overall organization to the distribution of GPCRs within neural compartments.

As discussed above, another important component impacting the physiologically plausible distance(s) between endogenous opioid peptide release location and responding receptor site is enzymatic degradation of the peptides. It is clear that aminopeptidase B and endopeptidase-24.11 have strong regulatory control over peptide signaling because in experiments where these enzymes are blocked not only is the manipulation sufficient to change behavior *in vivo* but also to facilitate the detection of electrophysiological responses to endogenous opioid peptide release ex vivo (e.g., Bourgoin et al., 1986; Fournie-Zaluski et al., 1984; Waksman et al., 1985), described in the next Section. Thus it seems the distribution and organization of several elements of the endogenous opioid system at the subcellular level may be more consistent with models of relatively limited peptide diffusion away from release sites rather than broad diffusion events generating larger volumes of transmission (Fig. 3A).

There may be other cellular structures that target diffusion to nearby opioid receptors as well. For example, in the LC axon terminals labeled for LENK contain both small clear and large dense core vesicles along the periphery of the synaptic terminal. MORs were detected on dendrites lateral to the postsynaptic density, and astrocytic processes surround these synapses in an arrangement that might limit diffusion of LENK to nearby MOR containing dendrites (Bockstaele et al., 1996). Overall, though, patterns of proximity of opioid receptors to peptide release sites remain to be resolved.

## 7. Functional endogenous opioid peptide release *ex vivo*

A few researchers have been able to observe physiological neural responses that are blocked by opioid receptor antagonists, an indication that endogenous opioid peptides were released in these brain slice preparations. That said, in most *ex vivo* preparations detecting endogenous peptide release has been elusive. Successful studies provide some clues about the neural activity that drives release and the time course of the impact of this peptide release.

Early *ex vivo* extracellular recordings in the dentate gyrus of the hippocampus provided evidence for functional release of endogenous opioid peptides acting at the KOR (Drake et al., 1994; Simmons et al., 1995). Several seconds of high frequency electrical stimulus trains delivered to the hilus resulted in a depression of population spikes detected by field recordings in the granule cell layer. This effect reversed within 10 min of the stimulation and was mostly blocked by the selective KOR antagonist norBNI. The prolonged, high frequency stimulation applied in these experiments is consistent with the hypothesis that release of endogenous opioid peptides depends on extended burst firing.

Studies in other brain regions indicate that more moderate stimulation may be sufficient to drive release of endogenous opioid peptides. One neural population that seems to readily release endogenous opioid peptides is the magnocellular neurosecretory cells of the hypothalamus, where dynorphin is co-packaged into dense core vesicles with vasopressin (Watson et al., 1982). *Ex vivo* electrophysiology studies show that stimulus trains delivered to these neurons drive release of an endogenous opioid peptide that inhibits depolarizing after-polarizations (Brown et al., 2004; Brown and Bourque, 2004). The effect was seen with all train lengths tested, 10–100 pulses, although longer trains did produce stronger inhibitions. This effect is blocked by norBNI, but not the selective MOR antagonist CTOP, making it likely that dynorphin is being released and acting at KORs. The physiological response was augmented in the presence of peptidase inhibitors, demonstrating how strongly peptidases control endogenous opioid peptide signaling. Interestingly, the dynorphin induced inhibition recovers within 20 s of the stimulus train, a faster time course of effect than is usually conceptualized for neuromodulators.

A more recent finding of a somatodendritic effect of endogenous opioid peptides detected with slice electrophysiology was reported by Winters and colleagues (2017) in the intercalated neurons of the amygdala. Bath application of the peptidase inhibitors bestatin (leucine aminopeptidase, aminopeptidase B, and triamino peptidase inhibitor), captopril (angiotensin-converting enzyme inhibitor), and thiorphan (endopeptidase-24.11/neprilysin, CD10 inhibitor) was sufficient to drive an outward current that was reversed by either the selective MOR antagonist CTAP or the non-selective opioid receptor antagonist naloxone. This was consistent with ultrastructure evidence in the same study that MENK-containing terminals synapse onto the dendrites of the intercalated cells. The magnitude of these responses was augmented by electrical stimulation, intended to produce additional neural activity.

Endogenous opioid peptide effects on presynaptic terminals have also been observed with *ex vivo* whole cell electrophysiology. For instance, a study of presynaptic control of glutamate terminals in the dorsal striatum from Atwood et al. (2014) supports the model of rapid and limiting endopeptidase catabolism of released endogenous opioid peptides. In these experiments glutamatergic excitatory postsynaptic currents (EPSCs) were electrically evoked once every 20s, a relatively infrequent stimulation pattern. When bestatin, captopril, and thiorphan were applied to the slice for 5 min, evoked EPSC amplitudes decreased for tens of minutes. This effect was blocked by the non-selective opioid receptor antagonist naloxone. The effect of the peptidase inhibitors was also prevented by metabotropic glutamate receptor type 5 (mGluR5) blockade, suggesting the endogenous opioid peptide release that leads to this synaptic modulation requires activation of mGluR5. In separate experiments, bath application of MENK, LENK, or dynorphin A each inhibited evoked EPSC amplitudes, showing that the opioid receptors on these glutamate terminals are responsive to each of these peptides.

In a subsequent study in the NAc, Trieu and colleagues (2022) showed that captopril application was sufficient to cause an inhibition of electrically evoked EPSCs, specifically in *Drd1* MSNs. This effect was blocked by naloxone. They also directly measured endogenous opioid peptide released from brain slices by bath application of high external KCl for 20 min to depolarize cells then collecting perfusate for peptide detection with LC-MS/MS. This broad excitation increased the perfusate concentrations of MENK-RF, dynorphin A 1–8, dynorphin B, MENK, and LENK. Captopril selectively increased the concentration of MENK-RF. When *Drd2* MSNs that express *Penk* were stimulated with channelrhodopsin-2 (20 Hz, 10 min) MENK and LENK concentrations in the perfusate increased, but when captopril was applied concomitantly with the light stimulation, MENK-RF concentrations, but not MENK or LENK concentrations, increased. These observations are consistent with MENK-RF being released and subsequently cleaved into MENK by angiotensin-converting enzyme.

Several groups have pinpointed a role for endogenous opioid peptide release in a form of long term depression (LTD) of feedforward inhibition in the hippocampus (Leroy et al., 2017, 2021; Piskorowski and Chevaleyre, 2013). In CA2, vasoactive intestinal peptide-expressing (VIP) GABAergic interneurons synapse onto parvalbumin-expressing (PV) GABAergic interneurons that innervate pyramidal cells. Specific patterns of tetanic stimulation excite VIP neurons and drive depression of the output of PV neurons resulting in a potentiation of postsynaptic potentials in CA2 pyramidal neurons. Most VIP neurons in CA2 express *Penk* and vice versa (Blasco-Ibáñez et al., 1998), and DOR is mostly expressed in interneurons in the hippocampus (Commons and Milner, 1997). Application of the DOR antagonists naltrindole or ICI174864 during the tetanic stimulation prevented the depression of the GABAergic inputs as well as the overall potentiation of the postsynaptic potentials recorded in pyramidal neurons. Short hairpin mRNA knockdown of *Penk* broadly in CA2 or specifically in VIP neurons also eliminated this LTD, thus identifying the source of the endogenous opioid peptide driving these synaptic changes (Leroy et al., 2021). Behaviorally, blocking DOR or knocking down *Penk* in CA2 interferes with formation of social memory in mice (Leroy et al., 2017, 2021). While the exact identity of the released *Penk*-derived peptide is not known, these studies elegantly identified the neurons releasing the peptide, the identity of the target receptor on PV interneurons, and related this function to a key behavior.

Together these observations demonstrate some dynamic features of the endogenous opioid peptide system. Endogenous opioid peptides can be released with action potential activity patterns well within the range of those that have been observed with *in vivo* extracellular electrophysiology during common behavior assays. In fact, *ex vivo* experiments indicate that in at least some circumstances minimal action potential activity is required to drive peptide release. Enzymatic degradation strongly controls the diffusion of opioid peptides away from release sites, spatially limiting the population of opioid receptors they can activate. And, endogenously released peptides can have relatively dynamic actions in brain slices, with rapid onset and reversal of effect on the order of 10s of seconds after the termination of stimulation. One limitation to interpreting *ex vivo* electrophysiological observations of responses is that the perfusion bath is essentially an infinite sink for any peptides or other molecules that are released; this likely limits the maximum extracellular peptide concentration and accelerates the drop in peptide concentration after release in this preparation. Another consideration is that the electrical or optogenetic stimulation utilized in *ex vivo* techniques generate a great deal of synchrony across many separate axons or cell bodies. In many brain regions smaller subpopulations of neurons are active simultaneously during a given behaviorally relevant epoch, making this type of broad stimulation supraphysiological. Therefore these model experiments may generate an overestimate of peptide release and its physiological impact. Still, these *ex vivo* studies provide an important framework for thinking about what kinds of neural activity patterns are needed to produce endogenous opioid peptide release and the time course of receptor signaling resulting from such release events.

## 8. Treating alcohol use disorder by blocking endogenous opioid activity with Naltrexone

### 8.1. Clinical development and experience

Can this mechanistic neurobiological research inform our understanding of neuropsychiatric disorders or maladaptive behaviors? Because the opioid receptor antagonist NTX decreases alcohol consumption in humans and animals (Gonzales and Weiss, 1998; Herz, 1997; Mitchell et al., 2009) it is strongly hypothesized that a key contributor to alcohol’s rewarding effects is activation of the endogenous opioid system. As an antagonist, NTX has an effect when endogenous opioid peptides are released and activating opioid receptors. Although NTX was originally approved in 1984 to treat heroin addiction, studies do not support its use for this indication (Coffa and Snyder, 2019; Greenstein et al., 1981; Julius and Renault, 1976 (eds); Lee et al., 2018; Minozzi et al., 2011; NRC, 1978; Rawson and Tennant, 1984). Oral NTX was approved for treatment of AUD in 1994 (Center for Substance Abuse Treatment, 2009).

Early studies investigating the efficacy of NTX were performed in conjunction with psychotherapy (Balldin et al., 2003; O’Brien et al., 1996; O’Malley et al., 1992; Oslin et al., 2008; Volpicelli et al., 1992) and found that NTX decreased alcohol craving, the frequency of return to alcohol consumption, and Addiction Severity Index (ASI) scores compared to placebo (O’Malley et al., 1992; Volpicelli et al., 1992). ASI evaluates the severity of the subject’s alcohol use, family, legal, social, psychological, and medical problems and was assessed at the beginning and end of the 12 week study (O’Malley et al., 1992). It is important to note that there was also an interaction between type of therapy and frequency of relapse: providing coping skills to patients in the treatment group proved best for relapse prevention (O’Malley et al., 1992). This is consistent with studies in which cognitive behavioral therapy improved AUD treatment outcomes (Ray et al., 2020). A six-month follow-up study showed that the NTX treated subjects were still less likely to drink heavily, suggesting ongoing efficacy (O’Malley et al., 1996).

A clue to how endogenous opioids contribute to the motivation to drink alcohol is in an early study where subjects reported that their alcohol induced “high” was diminished when on NTX (Volpicelli et al., 1992). This effect should decrease the motivation to continue drinking because it makes consumption less rewarding. In fact, and consistent with this, studies indicate that rather than promoting abstinence, NTX reduces heavy drinking episodes (O’Malley et al., 1992; Rohsenow, 2004).

One issue with oral NTX is subject non-compliance (Volpicelli et al., 1997), a common problem for substance use disorder treatments (Herbeck et al., 2005). To mitigate this, a long-acting intramuscular injection that lasts 4 weeks, brand named Vivitrol^®^, was developed (Center for Substance Abuse Treatment, 2009; Jarvis et al., 2018; O’Brien et al., 1996). In addition to compliance, another advantage of the injectable is more consistent blood levels of NTX compared to daily oral dosing (Johnson, 2007; Mannelli et al., 2014). Clinical trials for the long-acting injectable showed a 25% reduction in heavy drinking episodes compared to placebo (Garbutt et al., 2005). For patients that were abstinent before treatment began, the time to first drink with extended-release treatment was 41 days versus 12 days for those who received placebo (O’Malley et al., 2007). Importantly, only 14% of subjects discontinued treatment (Garbutt et al., 2005).

While duration of abstinence had long been used as the main clinical endpoint for AUD treatment studies, there is a mismatch between this endpoint and the model of NTX target action (Garbutt et al., 2005; Leighty and Ansara, 2019; O’Malley et al., 2007; Pettinati et al., 2011). That is, if the hypothesized mechanism is correct, NTX should affect signaling during drinking that causes endogenous opioid peptide release, not decrease the desire to drink while abstinent. Consistent with this model, oral NTX decreased total drinking days per month, decreased heavy drinking days per month, and increased days until the first heavy drinking event (O’Malley et al., 2007; Pettinati et al., 2011). Extended release NTX also decreased the number of heavy drinking days for patients with high severity AUD (Espiridion, 2019; Pettinati et al., 2011). Fewer heavy drinking days can improve mental and physical health, mitigating many of the problems associated with AUD.

Several side effects of oral and intramuscular NTX have been recorded, including drowsiness (30%), nausea (26%), vomiting (17%), decreased appetite (18%), abdominal pain (16%), insomnia (16%), dizziness (12%), and injection site reactions for intramuscular NTX (Jonas et al., 2014; Kranzler and Soyka, 2018). Nausea was less prevalent in patients with lower severity drinking episodes and longer abstinence before treatment initiation, consistent with NTX producing some opioid withdrawal by reversing endogenous opioid peptide actions (O’Malley et al., 2000).

It is important to note that most of these clinical data were collected in men (Schick et al., 2020), especially because there are known sex differences in opioid systems (Becker and Chartoff, 2019) and in motivation(s) to drink (Peltier et al., 2019). One study reported that NTX was not efficacious in heavy drinking women (Hernandez-Avila et al., 2006), but other studies report conflicting results (Baros et al., 2008; Peltier et al., 2019; Yoon et al., 2016). The COMBINE study, which is the largest study of pharmacotherapy treatment for AUD, did not detect any sex differences in NTX efficacy (Greenfield et al., 2010). Given data available now, it seems that sex differences do not limit NTX efficacy in treating AUD.

### 8.2. Current understanding of naltrexone function

A great deal of preclinical work has been done to determine how NTX works at the biological level to decrease alcohol consumption. Like with humans, NTX decreases alcohol consumption in other species (Bienkowski et al., 1999; Egli, 2005; Mitchell et al., 2009; Stromberg et al., 1998). Some differences in efficacy have been noted, such as sex differences in rats (Matzeu et al., 2018), and the pattern of alcohol access and route of NTX administration matters (Goodwin et al., 2001; Korpi et al., 2017). Animal studies suggest that consistent use of NTX changes the endogenous opioid system to make it less effective (Yoburn et al., 1989; Zukin et al., 1982), a potential consideration for intramuscular NTX.

Animal studies have also identified some of the brain regions that likely contribute to NTX’s efficacy. For instance, microinjection of NTX into the NAc decreases EtOH self administration and prevents the consumption induced increase in extracellular dopamine in the NAc (Gonzales and Weiss, 1998). Microinjection of the selective MOR antagonist CTOP into the VTA decreases EtOH consumption in rats (Margolis et al., 2008). NTX’s blockade of KOR actions may also contribute to its ability to decrease alcohol consumption. Systemic administration of selective KOR antagonists decreases EtOH consumption in rodents that are EtOH dependent or that have been subjected to an aversive stressor (Anderson et al., 2016; Rose et al., 2016; Sperling et al., 2010), although not in rodents that are neither dependent nor stressed (Mitchell et al., 2005). In EtOH dependent rodents, microinjecting a selective KOR antagonist specifically into the BNST (Erikson et al., 2018) or CEA (Kissler and Walker, 2016) decreases EtOH consumption. The contribution specifically of the KOR system to the motivation to consume alcohol is reviewed in (Anderson and Becker, 2017).

Microdialysis has been used to investigate the identities of the endogenous opioid peptides released in response to EtOH, the actions of which NTX would reverse. For instance, acute i.p. injection of EtOH leads to an increase in β-endorphin, MENK, and dynorphin A 1–8 in the NAc (Marinelli et al., 2003, 2005, 2006). A similar study in the VTA found an increase in β-endorphin, but not MENK following i.p. EtOH (Jarjour et al., 2009). One limitation of these studies that prevents making a link between endogenous opioid peptide release and the motivation to drink is the animals were not voluntarily consuming EtOH. Another is that i.p. EtOH injections generate discomfort. These were also first EtOH exposures for the study animals, and future studies are required to determine if similar changes in peptide levels occur with experienced drinkers or animals that have learned to associate the i.p. injection with the subsequent rewarding effects of EtOH.

Human imaging studies have also identified changes in brain activity induced by NTX in alcohol-using and abstinent individuals. Changes in fMRI BOLD signals do not indicate where NTX is binding, but provide important information on which brain regions might be contributing to human experience and behavior. BOLD signals often interact with the personality trait locus of control, a measure of an individual’s conceptualization of how much control one has over his/her life, with internal locus of control indicating a feeling of strong control. In alcohol using males, NTX modulated the brain-wide functional connectivity of rostral anterior cingulate cortex/ventromedial prefrontal cortex, enhancing connectivity of a left frontoparietal network and decreasing connectivity with visual and motor regions (Elton et al., 2019). The strongest changes with NTX were in individuals who indicated they consumed alcohol to cope (Elton et al., 2019). On placebo, those who showed stronger functional connectivity between these regions also had a more external locus of control (Elton et al., 2019). These studies start to build an understanding of the relationships between NTX effect, personality traits, and functional connectivity that may help predict who will respond to NTX and what kinds of changes in brain activity may be important for therapeutic effect.

A positron emission tomography (PET) study in humans does indicate endogenous opioid peptides are released in the NAc following alcohol consumption (Mitchell et al., 2012). Using PET detection of the highly selective MOR agonist [^11^C]carfentanil, Mitchell et al. (2012) found a decrease in radioligand binding in the NAc and orbitofrontal cortex after subjects consumed an alcoholic beverage. This was observed both in heavy drinkers and controls. There was also some indication that the magnitude of orbital frontal endogenous opioid peptide release relates to heavy drinking as the change in binding correlated with alcohol induced subjective high and several measures of problem drinking.

In humans, plasma levels of β-endorphin are increased during acute heavy intoxication, but in chronic drinkers the levels are lower than average (Zalewska-Kaszubska and Czarnecka, 2005). This low β-endorphin in people with AUD persists for more than ten years during abstinence (Arbol et al., 1995). Thus heavy or chronic alcohol consumption produces long term endogenous opioid alterations.

Risk of elevated alcohol use is a partially heritable trait in both humans and animals, and the endogenous opioid system can contribute (Edwards et al., 2018; Mitchell et al., 2012; Palmer et al., 2019; Schuckit et al., 2005; Verhulst et al., 2015; Walters et al., 2018). Humans with a family history of AUD tend to require higher blood alcohol levels to reach intoxication (Eng et al., 2005; Schuckit et al., 2004). Family history also interacts with NTX effects. In college students with a positive family history for alcohol abuse, there is a strong relationship between locus of control and the change in impulsive choice ratio in a delay discounting task, such that individuals with a strong internal locus of control showed an increase in impulsive choice on NTX while those with a strong external locus of control showed a decrease in impulsive choice on NTX (Altamirano et al., 2011). A similar pattern was found in abstinent alcoholics (Mitchell et al., 2007). Decreasing impulsive choice should have a beneficial effect with respect to decreasing alcohol consumption, and this work indicates a link to family history for this specific effect.

One interesting genetic polymorphism is A118G in OPRM1. This polymorphism is not associated with AUD, but is associated with NTX’s effectiveness (Anton et al., 2008; Bergen et al., 1997; Chamorro et al., 2012; Franke et al., 2001; Oslin et al., 2008); patients carrying the G allele had lower relapse rates and a longer time before returning to heavy drinking on NTX than those homozygous for the A allele (Chamorro et al., 2012; Oslin et al., 2008). Subjects with at least one copy of the G allele also had a significant reduction in the self-reported “high” due to alcohol intake while taking NTX compared to those lacking this allele (Ray and Hutchison, 2007). A transgenic mouse model containing this OPRM1 polymorphism was generated (Bilbao et al., 2015). Consistent with observations in humans, NTX selectively suppressed both home cage EtOH drinking and operant self-administration of EtOH in h/mOPRM1–118GG mouse line compared to the h/mOPRM1–118AA line (Bilbao et al., 2015). MORs with this polymorphism have an increase in binding affinity for β-endorphin 1–31, but no change in binding affinity for MENK, dynorphin A 1–17, or EM-1 (Bond et al., 1998). This SNP also appears to greatly decrease the number of MORs translated in a heterologous system (Zhang et al., 2005). The polymorphism reduces synaptic modulation by DAMGO and morphine (Popova et al., 2019; Robinson et al., 2015). While this polymorphism could lead to other kinds of biological differences in the endogenous opioid system *in vivo* such as changes in receptor distribution or signaling, so far findings suggest that β-endorphin 1–31 plays a particularly dominant role in the interaction between this SNP and NTX’s effects.

At least some of the differences that develop in rats bred for high and low EtOH consumption also reside in the opioid system. While NTX attenuates EtOH seeking in both alcohol preferring (P) and standard rats, P rats showed more sensitivity to low doses of NTX (Henderson-Redmond and Czachowski, 2014). A systemic EtOH injection increases β-endorphin release in the NAc in alcohol preferring but not alcohol-avoiding rats (Lam et al., 2009). In mice that consume high levels of EtOH (C57BL/6) a similar phenomenon has also been observed. There was more β-endorphin release in the hypothalamus after EtOH injection in C57BL/6 mice compared to alcohol averse DBA/2 mice (Waele and Gianoulakis, 1993).

Animal studies have also revealed some possible limitations to the efficacy of systemic administration of a non-selective opioid receptor antagonist like NTX. For instance, Kemppainen et al. (2012) showed that microinjecting the selective MOR antagonist CTOP into the ventral pallidum increases EtOH consumption and microinjection of the selective MOR agonist DAMGO decreases EtOH consumption, thus there is at least one brain site where blocking MOR opposes the desired effect. Also, microinjecting the selective DOR antagonist TIPP-Ψ into the VTA increases EtOH consumption (Margolis et al., 2008). DOR blockade is also anxiogenic (Balerio et al., 2005; Narita et al., 2006; Solati, 2011; Solati et al., 2010), which could be problematic, especially in individuals who drink alcohol to relieve anxiety. Therefore an opioid receptor antagonist that does not bind to DOR might have improved efficacy for AUD.

NTX does not bind to the nociceptin receptor, however in preclinical studies there is evidence that endogenous nociceptin release also contributes to the motivation to consume EtOH. The nociceptin receptor antagonist LY2940094/BTRX-246040 has proven safe in humans and in a small proof-of-concept clinical study decreased MDD symptoms, reduced heavy alcohol drinking, and increased abstinence (Witkin et al., 2019). In samples from humans with AUD, *Pnoc* mRNA expression levels in the hippocampus were 1.7 fold lower compared to controls (Kuzmin et al., 2009). In rodents, systemic blockade of nociceptin receptors decreases EtOH drinking (Brunori et al., 2019) and stress-induced reinstatement of EtOH seeking (Borruto et al., 2021). Microinjections of a nociceptin receptor antagonist into the VTA or the CEA, but not in the NAc, also decreases stress induced EtOH seeking (Borruto et al., 2019, 2021; Rorick-Kehn et al., 2016). Together these observations make blockade of the nociceptin receptor a provocative therapeutic target for AUD.

These are just some of the observations that help to understand how NTX decreases alcohol consumption. While NTX remains the most effective treatment for AUD, side effects are not rare and not everyone shows improvement. Another drawback of NTX is its ability to inhibit Cytochrome P450 (CYP), which is an important enzyme in the metabolism of opioids and other drugs, including those that treat other mental health disorders (AlRabiah et al., 2018; Smith, 2009). There is also evidence that the inactive NTX isomer, (+)-naltrexone, is a toll-like receptor 4 (TLR4) antagonist (Zhang et al., 2018). TLR4 antagonism could potentially have anti-inflammatory effects, but the benefits or drawbacks of this interaction are not known. Together these studies also identify several ways NTX might be improved upon to increase efficacy and decrease side effects.

## 9. Critical questions and conclusions

There are many remaining questions about endogenous opioid peptide function. Very little is known regarding when and where endogenous opioid peptides are released. Additional questions that require study include: how is peptide release driven, how far do the peptides diffuse from a release site, what is the extracellular concentration of peptide produced by release, how long does released peptide impact preand postsynaptic function, which signaling pathways are activated by the different endogenous opioid peptides at each opioid receptor, and do these phenomena vary with brain region? Recent reviews describe advances and ongoing challenges in detecting endogenous opioid peptide fluctuations (Conway et al., 2022; Girven et al., 2022). New transgenic rodents continue to be created that enable careful investigations of endogenous opioid peptide circuits. Improved resolution and sensitivity of sequencing based assays enable detection of lower abundance RNA message identifying previously unknown expression patterns for opioid peptides and receptors. For recent and future single cell transcriptome studies, when the datasets are made public researchers can probe them for opioid system expression patterns. Recent advances in sequential re-staining and co-registration of cleared tissue that enable labeling of a dozen or more proteins in the same sample promise the opportunity to integrate detection of elements of the endogenous opioid system into a broader context (Hickey et al., 2022; Kim et al., 2022; Murray et al., 2015). Advances in electron microscopy and reconstruction techniques provide improved understanding of the 3 dimensional structures such as where the peptides are released and the available extracellular space for diffusion to their target receptors. New tools for imaging fluctuations in neuropeptide levels *in vivo* are being developed (e.g. Ding et al., 2019). At the systems level, many questions remain regarding the contributions of the endogenous opioid system to sex differences including in pain, AUD, and SUD, as well as in treatment efficacies for these and other disorders.

Still, much progress has been made in our understanding of endogenous opioid peptide generation and how the different peptides bind and activate each of the opioid receptors. The opioid field has a wealth of tool compounds as well that have been used to investigate the behavioral impact of activating or blocking different elements of this system, and the subsequent observations have generated many hypotheses not only for how to improve on NTX efficacy for treating AUD, but also how to interact with the endogenous opioid system in novel ways to treat other indications. In addition to applying recent discoveries to design better analgesics that activate MOR and MDD treatments that block KOR, one exciting possibility is to boost the activity of endogenous opioid peptides with a positive allosteric modulator of MOR. Blockade of endogenous opioid peptide catabolism *in vivo* does not cause respiratory depression, raising the possibility that a positive allosteric modulator at MOR will generate analgesia without causing respiratory depression (Boudinot et al., 2001; Fournie-Zaluski et al., 1984; Kayser et al., 1989). Preclinical studies indicate potential for this approach (Kandasamy et al., 2021). Provocatively, positive allosteric activity at MORs might be one of the mechanisms by which ketamine produces analgesia (Gupta et al., 2011; Petrocchi et al., 2019). The efficacy of a positive allosteric modulator requires endogenous opioid peptide activity at the orthosteric binding site of the receptor. Thus, continuing to investigate the conditions when different endogenous opioid peptides are released with new and exciting tools, along with new advances in understanding structure-activity relationships in the opioid systems, has the potential to not only generate exciting biological observations but also new therapeutic strategies for various clinical indications.

## Data Availability

No data was used for the research described in the article.

## Abbreviations:

CYP: Cytochrome P450
DOR: Delta opioid receptor
KOR: Kappa opioid receptor
MOR: Mu opioid receptor
ORL1: Opioid receptor-like 1
Nociceptin receptor
PENK: proenkephalin
PDYN: prodynorphin
POMC: proopiomelanocortin
PNOC: pronociceptin
LENK: leu-enkephalin
MENK: met-enkephalin
EM: endomorphin
PAG: periaqueductal gray
NTS: nucleus of the solitary tract
BNST: bed nucleus of the stria terminalis
NAc: nucleus accumbens
LC: locus coeruleus
CEA: central nucleus of the amygdala
VTA: ventral tegmental area
DMH: dorsal medial hypothalamus
PVH: paraventricular nucleus of the hypothalamus
VIP: vasoactive intestinal peptide
DRN: dorsal raphe nucleus
